# Comparison of Color Stability of a Monochromatic Resin Versus Bulk-Fill and Micro-Hybrid Resins

**DOI:** 10.4317/jced.62653

**Published:** 2025-05-01

**Authors:** Bruno Spigno-Paco, José Giancarlo Tozo-Burgos, Roger Calla-Poma, Marco Sánchez-Tito

**Affiliations:** 1 Research Group on Dental Biomaterials and Natural Products, Faculty of Health Sciences, Universidad Privada de Tacna, 23000 Tacna, Peru; 2 Faculty of Dentistry, Universidad Nacional Mayor de San Marcos, 15001 Lima, Peru

## Abstract

**Background:**

To compare the color stability of a monochromatic nano-hybrid resin, a Bulk-Fill nano-hybrid resin, and a micro-hybrid resin after exposure to three staining beverages.

**Material and Methods:**

A monochromatic nano-hybrid resin (Vittra APS Unique), a Bulk-Fill nano-hybrid resin (Filtek™ Bulk-Fill) and a micro-hybrid resin (Filtek™ Z250 XT) were used. Two hundred twenty-eight samples (8 mm x 2 mm discs) were manufactured. The samples were divided into four subgroups based on the type of staining beverage (Coffee, black tea, Coca-Cola). Distilled water was used as control. The initial color of the samples was recorded after 24 hours, as well as after 7 and 15 days of immersion. The color difference (ΔE) was calculated with the CIE2000 formula. The two-way ANCOVA test was used for the analysis, and the significance level was adjusted to 5%.

**Results:**

A statistically significant interaction between beverage and resin types on ΔE values was observed while controlling for immersion time (*p*<0.001). In the analysis of the adjusted means, coffee and black tea primarily influenced the color change (ΔE) in the monochromatic resin, with values of 7.93 ± 1.90 and 6.93 ± 2.06, respectively. However, the most notable change was seen in the Bulk-Fill resin when exposed to Coca-Cola, which resulted in a ΔE value of 24.41 ± 0.41.

**Conclusions:**

The monochromatic resin showed a greater color change than the micro-hybrid and Bulk-Fill resins when immersed in coffee and black tea. Conversely, the Bulk-Fill resin was more prone to discoloration when exposed to Coca-Cola.

** Key words:**Resin, Chromatic stability, Spectrophotometry, Color perception, Carbonated beverages, Coffee, Tea.

## Introduction

Since the introduction of nano-hybrid resins, restorative materials have significantly advanced in their mechanical and aesthetic properties, primarily due to the incorporation of nanoparticles within their organic and inorganic matrix (1).

The development of Bulk-Fill resins has enhanced clinical efficiency by allowing the use of bulk increments (2). These resins incorporate camouflage technologies that help to integrate the restorations more seamlessly with the natural appearance of the tooth (2,3). In addition, conventional nano-hybrid resins are defined by their very small filler particles, which are less than 100 nanometers in size (4).

To address the need to reduce clinical time and help clinicians accurately select the right shade for restorations, new monochromatic resins have been introduced. These resins can adjust to the color of the surrounding tooth due to precise control over the size of the restorations. Additionally, the filler particles in these resins enhance the replication of enamel and dentin characteristics (5). However, there needs to be more evidence regarding the optical properties of these materials.

The color stability of dental resins is crucial for the longevity of restorations (6). Over time, resin restorations can change color due to various factors, including exposure to light, temperature variations, and the consumption of food and beverages (6-8). Staining beverages such as coffee and carbonated beverages, can significantly impact the color stability of resins by staining the surface of restorative materials (9). When the surface of composites frequently comes into contact with these staining beverages, pigments can gradually accumulate, leading to unwanted color changes like yellowing or surface staining (8,9). Furthermore, some pigments can penetrate the resin structure, making them difficult to remove using conventional cleaning methods (9).

Zulekha *et al*. evaluated the clinical performance of monochromatic universal composite resin and nano-hybrid composite resin as aesthetic restorations in maxillary deciduous incisors. Their results showed that the color stability and retention of the monochromatic resin were comparable to those of the multi-tone resin at both six- and twelve-month intervals. Additionally, the study found that the stability and color retention of both types of resin decreased over time (10). Zhu *et al*. evaluated the color adjustment potential of a monochromatic composite resin compared to four multi-tone composite resins. They found that the color adjustment potential depended on the material type and the background color (11). Khayat compared the optical properties of conventional composite resin restorations with those of monochromatic ones. His findings revealed that the color of single-shade restorations varied based on the shade of the teeth. Additionally, the stability of this color was influenced by various pigmenting substances. However, no significant differences were found when comparing the single-shade restorations to traditional ones (12).

Thus, color stability depends mainly on the quality of the materials used and the application technique (11). Dental resins are designed to remain color-stable and resist fading over time. The filler particles within the resins create a structural color that ranges from red to yellow, depending on the color of the surrounding tooth (1). However, prolonged exposure to staining substances can generate negative changes in color stability (9). There are conflicting results in the literature regarding the color stability of these resins, indicating a need for further investigation into their properties.

Currently, there are various resins available on the market for aesthetic restorations in the anterior region. Filtek™ Z250 XT is a micro-hybrid resin with nanoparticles filled with zirconium and silica that guarantee high mechanical properties. Filtek ™ Bulk-Fill is a nano-hybrid resin and provides excellent strength, low wear for durability and improved aesthetic properties. On the other hand, Vittra APS Unique is composed by nano-spheroidal charges of zirconia that allow to obtain a composite with excellent mechanical properties and exhibit a phenomenon called the “Chameleon effect”, which refers to the ability of a material do combine a color similar to that of its surrounding structures (13).

Consequently, due to the different properties of these materials, the objective of this study was to compare the color stability of a monochromatic nano-hybrid resin, a Bulk-fill nano-hybrid resin, and a micro-hybrid resin after exposure to staining beverages.

## Material and Methods

- Study design, sample calculation, and ethical considerations

An *in vitro* experimental study was conducted. The sample size was determined using the G*Power software. The two-way ANCOVA test was used, with an effect size of 0.25, a significance level of 5%, a power of 0.8, six degrees of freedom, and 12 groups were considered. The total calculated sample size was 228, with each group consisting of 18 samples. This research received approval from the Research Ethics Committee of the Faculty of Health Sciences at the Private University of Tacna, under registration number FACSA-CEI/121-09-23.

-Preparation of samples

**Figure 1:**
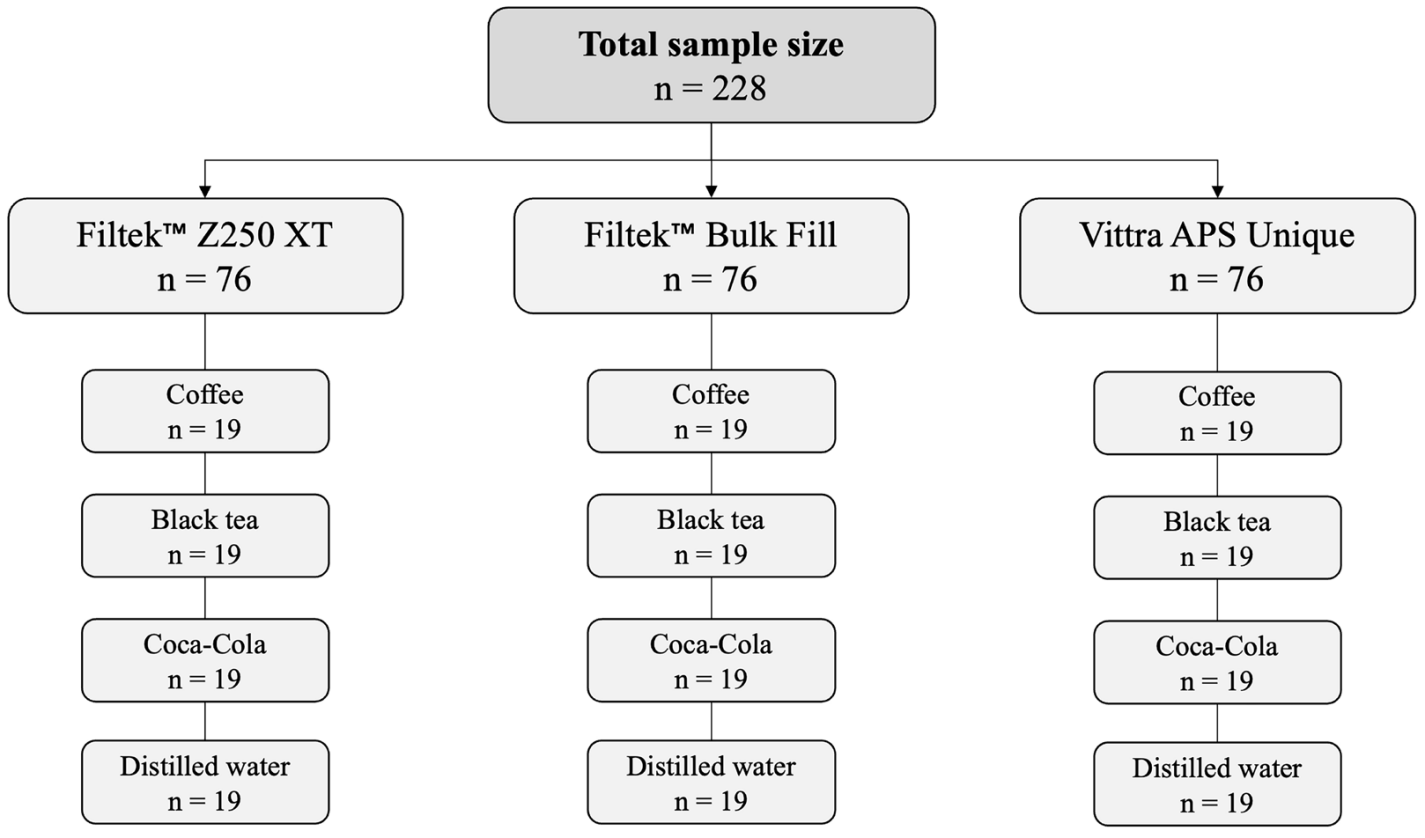
Distribution of study groups.

A total of 228 samples were prepared and allocated into 12 groups based on the type of resin and staining beverage (Fig. 1). The samples were made in a steel mold with a diameter of 8 mm and a thickness of 2 mm (8,14). The resins were packed into the molds in a single increment, a mylar strip was placed, and light pressure was applied to remove excess material. The samples were photoactivated using a LED lamp (VALO LED; Ultradent Products, South Jordan, UT, USA.) with an intensity of 1600 mW/cm² for 4 s. The samples were stored for 24 h before being polished with the Sof-Lex system (14). Finally, the samples were subjected to ultrasonic cleaning before the experiments and stored in distilled water at 37ºC until use. The characteristics of the resins used in this study are shown in  Table 1.

-Initial color measurement

The reference color (T1) of the samples was measured using a compact clinical spectrophotometer (VITA Easyshade Compact; VITA Zahnfabrik, Bad Sackingen, Germany). The tip of the spectrophotometer was placed perpendicular to the center of the sample. Color measurements were taken three times for each sample under standard D65 lighting conditions, in accordance with the CIELab system (10). The average of the three measurements was used to determine the reference color (T1).

-Immersion in staining beverages

The samples were randomly distributed in each group using the Research Randomizer Form 4.0 software (Social Psychology Network, Middletown, CT, USA). Each sample was placed individually in containers, where 10 mL of each beverage was added and kept at room temperature (14). The beverages were changed every 24 h. The coffee (Nescafé tradition, Nescafé®, Nestlé Brasil Ltda., Araras, SP, Brazil) was prepared by dissolving 4 g in 300 mL of boiling water (15). After 10 min, the solution was filtered through filter paper no. 1. The black tea (Herbi® pure tea, Lima, Peru) was prepared by immersing 4 bags in 300 mL of boiling water (16). Additionally, Coca-Cola (The Coca-Cola Company, Lima, Peru) was used as the third staining beverage. Distilled water served as a control.

-Color change records

**Figure 2:**
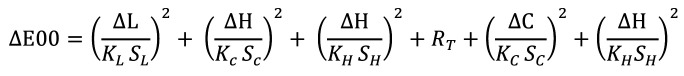
Formula.

The color of the samples was recorded after 7 (T2) and 15 (T3) days of immersion in the staining beverages. The color difference (ΔE) was calculated considering the initial value (T1) and the final value (T3) using the CIE2000 formula (10,17): (Fig. 2).

-Statistical analysis

The values from the control group were excluded from the analysis due to low data variability and their potentially inadequate effect on the model’s application. Data analysis was conducted using Stata® 18 software (StataCorp LP, College Station, TX, USA). A two-way ANCOVA was performed, incorporating the type of resin and staining beverage as variables, with immersion time included as a covariate. A profile plot was used to assess the magnitude of the interaction between the variables. The significance level was set at 5%.

## Results

 Table 2 shows the results of the two-way ANCOVA test, indicating that there was a statistically significant interaction between the type of resin and the type of staining beverages on the ΔE values while controlling for the immersion time (F = 2094.27, *p*<0.001). Additionally, 95.5% of the variability in the observed chromatic stability can be explained by the interaction between the resin types and the staining beverages (partial η² = 0.955).

**Figure 3:**
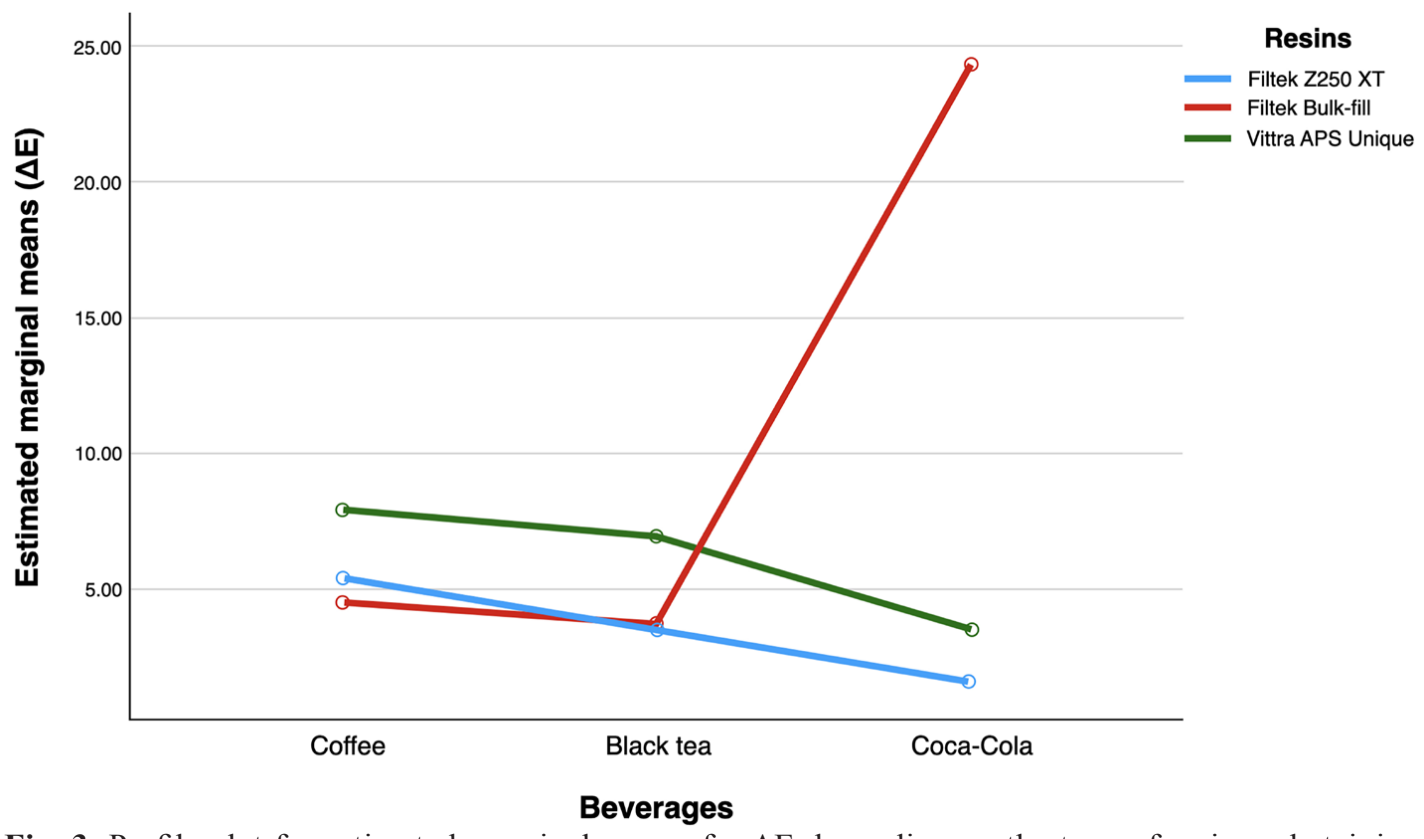
Profile plot for estimated marginal means for ΔE depending on the type of resin and staining beverages.

The pattern of lines displayed in Figure 3 indicates that there is an interaction effect between the two factors. Additionally, it is evident that the ΔE values were significantly higher for the Bulk-Fill resin when immersed in Coca-Cola, compared to the other types of resin.

Pairwise comparisons of adjusted mean differences for the main effects were conducted using Bonferroni-adjusted *p-values* ( Table 3). The results indicated that the differences in the adjusted means of the ΔE values were statistically significant among the three resins when they were immersed in coffee (*p*<0.001). When black tea was used as the staining beverage, no significant differences in the ΔE values were found between the Z250 XT and Bulk-Fill resins (*p*=1.000). However, there were statistically significant differences in the ΔE values between the monochromatic resin and both the Z250 XT and Bulk-Fill resins (*p*<0.001). Regarding the use of Coca-Cola as a staining beverage, significant differences in the adjusted means of the ΔE values among the three resins were also observed (*p*<0.001).

## Discussion

Chromatic stability is crucial for both the aesthetics and durability of dental restorations. This study aimed to explore how various staining beverages and immersion times affect the color stability of three different types of resins. The results indicated a significant interaction between the type of resin and the staining beverages, impacting the chromatic stability of the materials.

The results indicated that the Bulk-Fill resin exhibited the most significant change in color stability when exposed to Coca-Cola. Previous studies have demonstrated that Bulk-Fill resins are susceptible to color changes when in contact with pigmented substances. Shamszadeh *et al*. found that Bulk-Fill resin showed a greater variation in color stability compared to nanohybrid resins when immersed in coffee (8). Silva *et al*. evaluated three Bulk-Fill resins and a control group, demonstrating that the Bulk-Fill resin had greater pigmentation than the control group due to the presence of pigments and particle size (18). Bahbishi *et al*. evaluated the color stability of bulk-Fill resins when exposed to common beverages, comparing them to a universal resin (Z250 XT), which showed the greatest color stability against coffee (19). Bulk-Fill resins are designed to allow greater depth curing with a single application, offering improved translucency (18).

Furthermore, it has been suggested that a higher concentration of organic components in the resin leads to decreased resistance against water degradation and moisture absorption, resulting in lower color stability (20,21). Conversely, conventional nano-hybrid resins may appear opaquer due to their composition and particle size (1,14).

Serin-Kalay reported a significant difference in chromatic stability for Bulk-Fill resin compared to conventional resins when exposed to pigmentation with Coca-Cola (16). This effect can be attributed to the additional components found in the drink, such as caffeine. Coca-Cola also contains various additives, including carbonated water, sugar, phosphoric acid as an acidulant, and coloring (E-150d), among others (22). Barbosa and Cardoso studied how carbonated drinks impact the stability of resin compounds. Their findings indicated that both the type of beverage and the duration of immersion influenced the color stability of the resins. However, they found that inorganic fillers, as well as finishing and polishing methods, did not affect the color (23).

Prolonged exposure to pigment beverages can have an impact on surface roughness by promoting the detachment of inorganic fillers from the resin matrix leading to the formation of surface flaws, which increases susceptibility to external pigments (24). Particularly, prolonged exposure to phosphoric acid contained in Coca-Cola could promote the formation of defects, pores and loss of material on the surface, which would facilitate a progressive change in the color of the resin matrix (25). However, the results of Lopes-Rocha *et al*. showed that Coca-Cola had a smaller effect on the color change of Brilliant EverGlowTM, FiltekTM Supreme XTE and Admira Fusion® resins than when they were exposed to coffee and wine, after 40 hours of immersion (26). The authors argue that the presence of phosphate ions contained in Coca-Cola can block the corrosive effect of phosphoric acid. Thus, the differences observed between the results of this study and ours may be due to the immersion time periods, since in our study the immersion was for a maximum period of 15 days, demonstrating a significant interaction between the type of resin and the type of drink adjusted for the immersion time on the color changes.

Our study supports the idea that the interaction between resin type and staining beverages affects discoloration. This means that discoloration is not consistent across all combinations of resins and beverages. Some resin types are more prone to discoloration when exposed to specific beverages. Monochrome and Bulk-Fill resins have different chemical compositions, which influence their resistance to fading (2,12,16). As mentioned earlier, Bulk-Fill resins exhibit greater translucency due to clinical requirements and their organic matrix with nanometer sized particles (1–100 nm) compared to the Z250 XT resin with an average particle size of 0.6 µm (16,27) and Vittra APS Unique with nano-spheroidal charges of 200 nm (1,6).

Beverages like coffee, black tea, and Coca-Cola contain various types and concentrations of pigments that interact differently with resin surfaces (23,28,29). Controlling the immersion time indicates that the duration of exposure is a critical factor that can increase the level of discoloration. The results showed that both staining beverage and the type of resin significantly affect the chromatic stability (ΔE) of dental resins.

Different beverages can have varying effects on the discoloration of dental resin. Coffee is particularly known for causing noticeable discoloration due to its pigment compounds (23,25,28). Black tea also affects resin, as it contains tannins and other natural pigments that can penetrate and stick to the surface (30). Similarly, Coca-Cola contributes to discoloration through its artificial colors and acids (23). This could be the result of an adsorption and absorption process of substances with lower polarity, such as the yellow pigments contained in coffee and other beverages, which have the ability to penetrate deeper layers of the resin matrix (31).

Monochromatic resins have the ability to emulate the structural color of the surrounding tooth, controlled by the filler particle size (1,5,6). This characteristic makes them adapTable and versatile for achieving dental aesthetics. Truong *et al*. (32) assessed the ability of these resins to adjust and mix colors, concluding that monochromatic resin is more effective in the incisal and occlusal thirds compared to other areas.

All the resins studied exhibited significant differences in ΔE values when exposed to coffee, indicating a notable impact on the discoloration of dental resins, regardless of the resin type.

In our study, the Bulk-Fill resin demonstrated greater susceptibility to discoloration, largely due to its increased translucency and particle size, especially when exposed to Coca-Cola. Additionally, the duration of exposure to these staining beverages was a crucial factor affecting the extent of discoloration. Prolonged immersion time resulted in increased discoloration, highlighting that both the type of resin and the length of contact with the beverages are essential for maintaining color stability.

One of the main limitations of this *in vitro* study is that laboratory conditions do not fully replicate the oral environment to which the resins are exposed. This discrepancy could impact the applicability of the findings. Further research is needed to include other variables that could influence the results of this investigation such as surface roughness, polishing techniques, the nature of the staining agents that may influence the color stability of the resins, the application time of the staining agents. Additionally, it is necessary to conduct studies that assess other optical properties of the monochromatic resins such as translucency and gloss. Future clinical studies could be applied to confirm the results of the *in vitro* studies.

## Conclusions

The evaluated dental resins exhibited different levels of color stability based on the type of resin and the staining beverage used. The monochromatic resin (Vittra APS Unique) showed a greater color change than the micro-hybrid resins (Filtek™ Z250 XT) and Bulk-fill (Filtek™ Bulk-Fill) when immersed in coffee and black tea. Additionally, the Bulk-Fill resin was particularly prone to discoloration when exposed to Coca-Cola.

## Figures and Tables

**Table 1:** Characteristics of the resins used in the study.

Composite	Color	Manufacturer	Type	Composition	Filler (wt/vol%)	Particle size
Filtek™ Z250 XT	A1	3M ESPE, USA	Micro-hybrid	Bis-GMA, Bis-EMA, UDMA, TEGDMA, silane treated ceramic filler, silane treated silica filler	78/60	0.4 µm – 1.0 µm
Filtek ™ Bulk Fill	A1	3M ESPE, USA	Nano-hybrid	Ceramics treated with silane, UDMA, aromatic Dimethacrylate Urethane, Silica treated with silane, ytterbium fluoride, DDMA, Zirconia treated with silane, Water, Monomer AFM-1, EDMAB, Benzotriazole, Dioxide of titanium	76.5/58.4	20 nm silica filler, 4-11 nm zirconia filler
Vittra APS Unique		FGM, Dental Group, Joinville, Brazil	Nano-hybrid	Mixture of methacrylate monomers, UDMA, TEGDMA, photoinitiator compound (APS), boron-aluminum-silicate glass	72-80/52-60	

**Table 2:** Two-way ANCOVA.

Source	Sum of squares	Df	Mean square	F	Sig.	η^2^ partial
Corrected model	17347.805	9	1927.534	1429.844	<0.001	0.970
Intersection	4542.493	1	4542.493	3369.621	<0.001	0.895
Immersion time	341.141	1	341.141	253.058	<0.001	0.390
Staining Beverage	1908.699	2	954.349	707.936	<0.001	0.782
Type of resin	3805.034	2	1902.517	1411.287	<0.001	0.877
Beverage*Resin	11292.931	4	2823.233	2094.274	<0.001	0.955
Error	532.489	395	1.348			
Total	36707.209	405				

Df: degree of freedom.

**Table 3:** Adjusted means for ΔE according to intervention groups.

Staining Beverage	Type of resin	Mean_aj_^*^	Standard deviation	95% confidence interval	
				Lower bound	Upper bound
Coffee	Z250 XT	5.33^a^	1.85	4.99	5.67
	Bulk-Fill	4.48^b^	1.31	4.14	4.82
	monochromatic	7.93^c^	1.90	7.59	8.27
Black tea	Z250 XT	3.52^d^	1.67	3.18	3.86
	Bulk-Fill	3.72^d^	1.77	3.38	4.06
	monochromatic	6.93^e^	2.06	6.59	7.27
Coca-Cola	Z250 XT	1.52^f^	0.45	1.18	1.86
	Bulk-Fill	24.41^g^	0.41	24.07	24.75
	monochromatic	3.49^h^	0.68	3.15	3.83

* The mean values of the groups for the independent variables were adjusted by the covariate Immersion time. Different superscript letters for each type of pigment drink indicate statistically significant differences at a p value adjusted by Bonferroni (*p*< 0.0167).

## Data Availability

The datasets used and/or analyzed during the current study are available from the corresponding author.
